# Adherence to basic hygiene routines in an infectious disease setting: associations with knowledge and professional role

**DOI:** 10.3389/fpubh.2026.1920433

**Published:** 2026-09-08

**Authors:** Greta Nykvist, Moa Nilsson, Katarina Bohm, Catarina Nahlén Bose

**Affiliations:** 1 Department of Infectious Diseases, Karolinska University Hospital, Stockholm, Sweden; 2 Department of Medicine, Ward 4, Norrtälje Hospital, Tiohundra AB, Stockholm, Sweden; 3 Department of Health Sciences, The Swedish Red Cross University, Huddinge, Sweden

**Keywords:** cross infection, guideline adherence, hand hygiene, health personnel, infection control, knowledge

## Abstract

**Introduction:**

Healthcare-associated infections (HAIs) are a major global health concern, leading to increased morbidity, mortality, healthcare costs, and prolonged hospital stays. Adherence to basic hygiene routines is a key preventive measure, yet adherence varies among healthcare professionals.

**Objective:**

To examine adherence to basic hygiene routines among healthcare professionals in an infectious disease clinic and to explore how knowledge, profession, and work-environment factors influence adherence.

**Methods:**

A cross-sectional study using a quantitative observational and questionnaire-based design was conducted at an infectious disease clinic in Sweden. A total of 69 healthcare professionals (assistant nurses, registered nurses, and physicians) were recruited through convenience sampling. Data were collected over a four-week period through structured observations, a knowledge test, and a self-assessment questionnaire. The observations were conducted during day and evening shifts using a structured observation protocol. Statistical analyses included descriptive statistics, ANOVA, non-parametric tests, and correlation analyses.

**Results:**

The overall observed adherence to basic hygiene routines was 76.4% (±20.2). Registered nurses demonstrated significantly higher adherence than physicians. Self-assessed adherence was higher (92.1% ± 3.3), indicating a discrepancy between perceived and observed behavior. Knowledge levels were generally high (median 90.9%, IQR 81.8–97.7), with registered nurses scoring significantly higher than other professions. No significant correlation was found between knowledge and observed adherence. Perceptions of the impact of the work environment factors on hand hygiene varied, with substantial proportions reporting both agreement and disagreement.

**Conclusion:**

Although healthcare professionals demonstrated good knowledge and generally positive self-assessed adherence, observed adherence to hygiene routines was suboptimal and varied between professions. The lack of correlation between knowledge and adherence suggests that factors beyond knowledge, such as organizational culture and behavioral aspects, are critical. Targeted interventions focusing on behavioral change, feedback, and interprofessional collaboration are needed to improve adherence and reduce HAIs.

## 1 Introduction

A healthcare-associated infection (HAI) is defined as an infection that occurs during the course of healthcare or that is acquired within a healthcare setting and becomes apparent after discharge. The term also includes infections acquired by healthcare workers in the workplace (1).

Every year, a large number of patients are affected by HAIs, significantly impacting healthcare costs and prolonging hospital stays compared with patients admitted for the same primary diagnoses (2–4). Within Europe, HAIs are estimated to result in approximately 16 million additional hospital days and 37,000 deaths annually. These infections cost around USD 7 billion every year (1). Approximately 57,000 individuals are affected by HAIs in Sweden each year, with around 1,300 deaths attributed to these infections, and the costs are estimated at approximately SEK 8 billion (3).

Semmelweis and Nightingale emphasized the importance of hand hygiene in preventing the spread of infection as early as the nineteenth century (5, 6). Current evidence demonstrates that adherence to hygiene routines reduces the incidence of HAIs (7, 8). The most common route of transmission is through the hands of healthcare workers, which is considered the greatest risk for the spread of microorganisms (9). In 2009, the WHO developed global guidelines for hand hygiene in healthcare (10). Based on these guidelines, the Swedish National Board of Health and Welfare has developed regulations for basic hygiene routines. These routines extend beyond the hand hygiene practices described by the WHO to include regulations on work clothing, jewelry, bandages, and the use of aprons. All personnel who have physical contact with patients are, by Swedish law, required to adhere to these guidelines (11, 12).

Differences in adherence may be related to variations in educational background among healthcare professionals. Physicians undergo the longest and most extensive academic training, followed by registered nurses with a university education, while assistant nurses usually have upper secondary education. However, studies suggest that even among healthcare professionals with higher education, adherence to hygiene routines is not always optimal (13).

Moreover, studies have shown that other factors, such as insufficient knowledge, stress, high workload, time constraints, lack of motivation, and negative attitudes adversely affect adherence to hand hygiene (14–16). Education about hygiene routines, their importance, and the consequences of non-adherence has been shown to increase the consumption of hand disinfectant and improve compliance with hand hygiene (14, 17, 18). Factors such as patient contact, exposure to bodily fluids, prevention of cross-infection, and personal protection are associated with increased motivation and adherence to hand hygiene. In contrast, adherence to hand hygiene tends to be lower before clean tasks and aseptic procedures (13, 15, 19).

To the best of our knowledge, there is limited research on adherence to the comprehensive framework of basic hygiene routines, as existing research predominantly addresses knowledge and adherence related specifically to hand hygiene. Both globally and nationally, research has focused on hand hygiene. Although hand hygiene is a major component of basic hygiene routines, there are other aspects of basic hygiene that are equally important for preventing HAIs but have received considerably less attention. This highlights a gap in the current literature and underscores the need to investigate adherence to basic hygiene routines as a whole. Furthermore, there is a lack of studies conducted specifically within infectious disease clinics in Sweden. This setting is particularly important to investigate, as healthcare professionals in infectious disease clinics frequently encounter patients with transmissible infections. Consequently, this study aims to examine adherence to basic hygiene routines among healthcare professionals in a Swedish infectious disease clinic, as well as to explore how knowledge, work environment factors, and professions influence adherence. By focusing on the comprehensive framework of basic hygiene routines within this specific context, this study contributes new knowledge to an area where evidence remains limited.

## 2 Methods

### 2.1 Study design

This was a cross-sectional study using a quantitative observational and questionnaire-based design. The study was conducted at an infectious disease clinic at a university hospital in Sweden. At the time of data collection, the wards had a total of 43 hospital beds.

### 2.2 Sample

#### 2.2.1 Inclusion criteria

The inclusion criteria for this study encompassed assistant nurses, registered nurses and physicians.

#### 2.2.2 Non-inclusion criteria

Pre-enrollment non-inclusion criteria were students, paramedical professionals, ward assistants, agency staff and consulting physicians. Agency staff were not included because they were only intermittently present on the ward, which created a risk that they would not complete the study. Paramedical professionals were not included because there were too few individuals from these professional groups working at the clinic to present results at a group level. It would also have been difficult to ensure that their participation remained anonymous when reporting the results. Students were not included due to ethical concerns about exposing them to additional stress beyond their student role.

#### 2.2.3 Sample size

As the study was conducted in a single infectious disease clinic with a finite population of eligible healthcare professionals, all eligible staff members were invited to participate. Therefore, no formal *a priori* sample size calculation was performed. A *post hoc* sensitivity analysis indicated that, with 69 participants and a significance level of 0.05, the study had 80% power to detect correlations of approximately *r* = 0.33 or larger and ANOVA effects of approximately *η*^2^ = 0.11 or larger.

### 2.3 Data collection procedure

Participants were recruited using convenience sampling from all eligible healthcare professionals at the study site. Data were collected over a four-week period in February 2025 using structured observations, a knowledge test, and a self-assessment questionnaire. Only healthcare personnel who provided consent were included in the study. All employees were informed about the study via email prior to its commencement. Those who did not wish to participate were asked to notify the authors in advance. On the days when observations were conducted, employees were reminded at the beginning of their shift that observations would take place during the day and that participation was voluntary. Following each observation, the observed individual was informed that the observation had occurred and was asked whether they consented to participate in the study. Written informed consent was subsequently obtained from all participants who agreed to take part.

### 2.4 Observation of adherence to basic hygiene routines

Observations were registered using a previously developed protocol by the Swedish National Board of Health and Welfare (20). Minor adjustments included distinguishing between three separate instances in which hand sanitizer should be used: before and after the use of gloves; before and after patient contact; and before and after contact with objects in the patient’s immediate environment. The adjustments were made to obtain more specific data. The observation protocol comprised 13 items, of which four assessed participants’ adherence to the dress code regulations. Specifically, these items evaluated compliance with the required clothing, adherence to policies regarding nail length and the absence of nail polish, the absence of jewelry and bandages on forearms and hands, and whether hair and beards were appropriately tied back. The remaining nine items evaluated participants’ adherence to hand disinfection practices and the correct use of gloves and aprons. Specifically, these items assessed whether hand disinfectant was used before and after patient contact, before putting on and after removing gloves, and before and after contact with the patient’s immediate surroundings. In addition, the protocol recorded whether hand washing was performed when indicated and whether gloves and aprons were used when indicated. Each correctly performed procedure was assigned one point, whereas incorrectly performed procedures received no points. If an item was not applicable, this was recorded in the observation protocol.

The observations were carried out during a patient encounter, from the time the participant entered the patient room until they left the room. The observations were conducted outside the patient rooms, with visibility through two windows. Patient encounters that took place in bathrooms or shower rooms were not observed. Details such as whether hand disinfectant was rubbed until dry and whether gloves were removed correctly were not part of the protocol and were not observed.

The observations were conducted independently by two observers during both day and evening shifts. Each observer performed the observations separately using the same observation protocol. The observers worked closely together throughout the data collection process, and any observations considered uncertain or ambiguous were discussed to reach a consensus and ensure accurate documentation. All observations were recorded using a pre-printed, paper-based observation protocol.

### 2.5 Knowledge test and self-assessment questionnaire

After the observation, participants were asked to complete a knowledge test and a self-assessment questionnaire developed by Johansson and Björk (21). The questionnaire was adjusted to reflect the current regulations of the Swedish National Board of Health and Welfare. It consisted of five parts: (1) education and knowledge acquisition, (2) single-item self-assessed adherence, knowledge and importance of knowledge to basic hygiene routines, (3) knowledge test (Cronbach’s alpha [*α*] = 0.813), (4) self-assessment of adherence to basic hygiene routines scale (*α* = 0.734) and (5) work-environment scale (*α* = 0.796). Education and knowledge acquisition comprised four items addressing participants’ training in basic hygiene routines during their professional education and in the workplace, whether their knowledge was regularly updated, and how such knowledge was acquired. In the three single-item questions, participants rated their knowledge of basic hygiene routines, the perceived importance of knowledge about these routines, and the extent of their own adherence on a three- to four-point Likert scale. The knowledge test consisted of 11 multiple-choice questions assessing knowledge of basic hygiene routines. The questions contained between three and five answer options, of which more than one answer could be correct. Points were awarded for both correctly selected and correctly omitted answer options, with a maximum score of 44 points. Part four included a self-assessment of adherence, where participants rated their adherence to specific components of basic hygiene routines using a 4-point Likert scale indicating the extent to which they agreed with each statement. It consisted of 12 items, however, one item (“I am more strict with hand hygiene when the patient has a known infection, e.g., MRSA, ESBL”) was excluded from the total adherence score because psychometric evaluation indicated that it did not perform consistently with the remaining items. Part five comprised a work-environment scale, in which participants evaluated how various work environment factors influenced their adherence to hand hygiene using a 4-point Likert scale.

### 2.6 Statistical analysis

The data were analyzed using IBM SPSS Statistics 30.0. Missing values were replaced using mean imputation. Percentage normalization was used for the total score on the observation protocol as the maximum score depended on the number of applicable tasks. The data were screened and departure from normal distribution was identified in three items: the total score on the knowledge test (skewness = −1.695, kurtosis = 3.337), the single-item question on the importance of having knowledge of basic hygiene routines in healthcare (skewness = −2.453, kurtosis = 4.136) and one item in the work-environment scale that self-assessed whether participants aligned their own hand hygiene behavior with that of their colleagues (skewness = −1.288, kurtosis = 0.779). Non-parametric tests were applied for these variables, where applicable. All other items had acceptable values for normality (skewness ranging from −1.004 to 0.580 and kurtosis ranging from −1.785 to 0.779) and were analyzed using parametric statistics.

To examine differences in education about basic hygiene routines, regular updates, and knowledge acquisition between professions Chi-square tests or Fisher’s exact tests (applied when the expected cell counts were less than five) were performed. Descriptive statistics are presented as frequencies and percentages. Differences in knowledge and adherence to basic hygiene routines between professions were analyzed using one-way ANOVA with Bonferroni post-hoc tests. For non-normally distributed variables, Kruskal–Wallis tests with pairwise comparisons were performed. Correlations between variables were analyzed using Pearson’s or Spearman’s correlation coefficients, denoted by *r* and *r_s,_* respectively. The significance level was set at *p* < 0.05.

## 3 Results

### 3.1 Background characteristics

A total of 69 healthcare personnel participated in the study with the distribution of 27 assistant nurses, 29 registered nurses and 13 physicians. Almost all (98.6%) had received education about basic hygiene routines during their professional education, including 100% of assistant nurses, 100% of registered nurses, and 92.3% of physicians. No statistically significant differences were found between the professions; both assistant nurses and registered nurses achieved 100% training rates, and neither differed significantly from physicians (Fisher’s exact test, *p* = 0.325 for assistant nurses vs. physicians, and *p* = 0.310 for registered nurses vs. physicians).

Regarding training at the current workplace, 73.9% of the total sample had received such education. No significant differences were found between the professions regarding workplace education: 85.2% of assistant nurses, 69.0% of registered nurses, and 61.5% of physicians reported having received such training (registered nurses vs. physicians: *χ*^2^(2) = 0.87, *p* = 0.649; assistant nurses vs. registered nurses: *χ*^2^(2) = 2.41, *p* = 0.299; assistant nurses vs. physicians: *χ*^2^(2) = 4.40, *p* = 0.111).

Overall, 81.2% of the participants regularly updated their knowledge on basic hygiene routines. This proportion was 92.6% among assistant nurses, 82.8% among registered nurses, and 53.8% among physicians. Assistant nurses reported updating their knowledge significantly more often than physicians (Fisher’s exact test, *p* = 0.008, *φ* = 0.45). No significant differences were found between registered nurses and physicians (Fisher’s exact test, *p* = 0.066) or between assistant nurses and registered nurses (Fisher’s exact test, *p* = 0.424).

The most common ways of retrieving information were via the Handbook for Healthcare (32.6%) and internal education at the hospital department (19.7%). Significant differences in specific knowledge acquisition methods were found between the professions. The Handbook for Healthcare was used by 74.1% of assistant nurses and 82.8% of registered nurses, which was significantly higher than the usage rate of 30.8% among physicians (registered nurses vs. physicians: Fisher’s exact test, *p* = 0.003; assistant nurses vs. physicians: *χ*^2^(1) = 6.86, *p* = 0.009). Assistant nurses and registered nurses did not differ significantly in their use of the Handbook for Healthcare (*χ*^2^(1) = 0.63, *p* = 0.429). Internal education was utilized by 70.4% of assistant nurses, which was significantly higher than the usage rates among registered nurses (20.7%; *χ*^2^(1) = 13.96, *p* < 0.001) and physicians (30.8%; *χ*^2^(1) = 5.63, *p* = 0.018), while registered nurses and physicians did not differ significantly (Fisher’s exact test, *p* = 0.697). Furthermore, the hospital’s local PM was used by 61.5% of physicians and 51.7% of registered nurses, which was significantly higher than the usage rate of 14.8% among assistant nurses (registered nurses vs. assistant nurses: *χ*^2^(1) = 8.50, *p* = 0.004; physicians vs. assistant nurses: Fisher’s exact test, *p* = 0.008). Physicians and registered nurses did not differ significantly in their use of local PM guidelines (*χ*^2^(1) = 0.35, *p* = 0.555). Contact with infection prevention and control units was reported by 25.9% of assistant nurses and 23.1% of physicians, whereas 0.0% of registered nurses utilized this method (assistant nurses vs. registered nurses: Fisher’s exact test, *p* = 0.004; physicians vs. registered nurses: Fisher’s exact test, *p* = 0.025; assistant nurses vs. physicians: Fisher’s exact test, *p* = 1.000). More details are presented in Table 1.

**Table 1:** Participants’ education and knowledge acquisition.

Variable	Total % (n)	Assistant nurse	Registered nurse	Physician
1. Education on basic hygiene routines:				
- During professional education	98.6% (68)	100% (27)	100% (29)	92.3% (12)
- At current workplace	73.9% (51)	85.2% (23)	69% (20)	61.5% (8)
2. Regularly update knowledge on basic hygiene routines	81.2% (56)^b^	92.6% (25)	82.8% (24)	53.8% (7)
3. Knowledge acquisition via:	147 responses in total	62 responses	63 responses	22 responses
- The hospital’s local PM	18.4% (27)^a,b^	6.5% (4)	23.8% (15)	36.4% (8)
- The Handbook for Healthcare	32.6% (48)^b,c^	32.3% (20)	38.1% (24)	18.2% (4)
- Contact with Infection prevention and control	6.8% (10)^a,c^	11.3% (7)	–	13.6% (3)
- Asking colleagues	17.7% (26)	19.4% (12)	19.0% (12)	9.1% (2)
- Internal education	19.7% (29)^a,b^	30.6% (19)	9.5% (6)	18.2% (4)
- Others	4.8% (7)^a^	–	9.5% (6)	4.5% (1)

^a^*p* < 0.05 difference between assistant nurse and registered nurse, ^b^*p* < 0.05 difference between assistant nurse and physician, ^c^*p* < 0.05 difference between registered nurse and physician.

Percentages represent the proportion of participants (total *n* = 69) in question 1 and 2 and the proportion of responses (total *n* = 147) in question 3 (multiple choices allowed).

### 3.2 Adherence to and knowledge about basic hygiene routines

The overall mean adherence to basic hygiene routines across all observations was 76.4% (SD = 20.2%), calculated as a proportion of the maximum possible adherence score. A one-way ANOVA revealed a significant main effect of profession on observed adherence scores, *F*(2, 66) = 3.28, *p* = 0.044, *ɳ*^2^ = 0.091. Post-hoc pairwise comparisons using the Bonferroni correction indicated that registered nurses (*M* = 81.9%, SD = 16.8%) demonstrated significantly higher adherence than physicians (*M* = 62.2%, SD = 30.5%) with a mean difference of 16.65% (95% CI [0.65, 32.65]), adjusted *p* = 0.039. No statistically significant differences in adherence were found between assistant nurses (*M* = 75.8%, SD = 15.3%) and registered nurses (*p* = 0.749) or between assistant nurses and physicians (*p* = 0.339).

**Figure 1:**
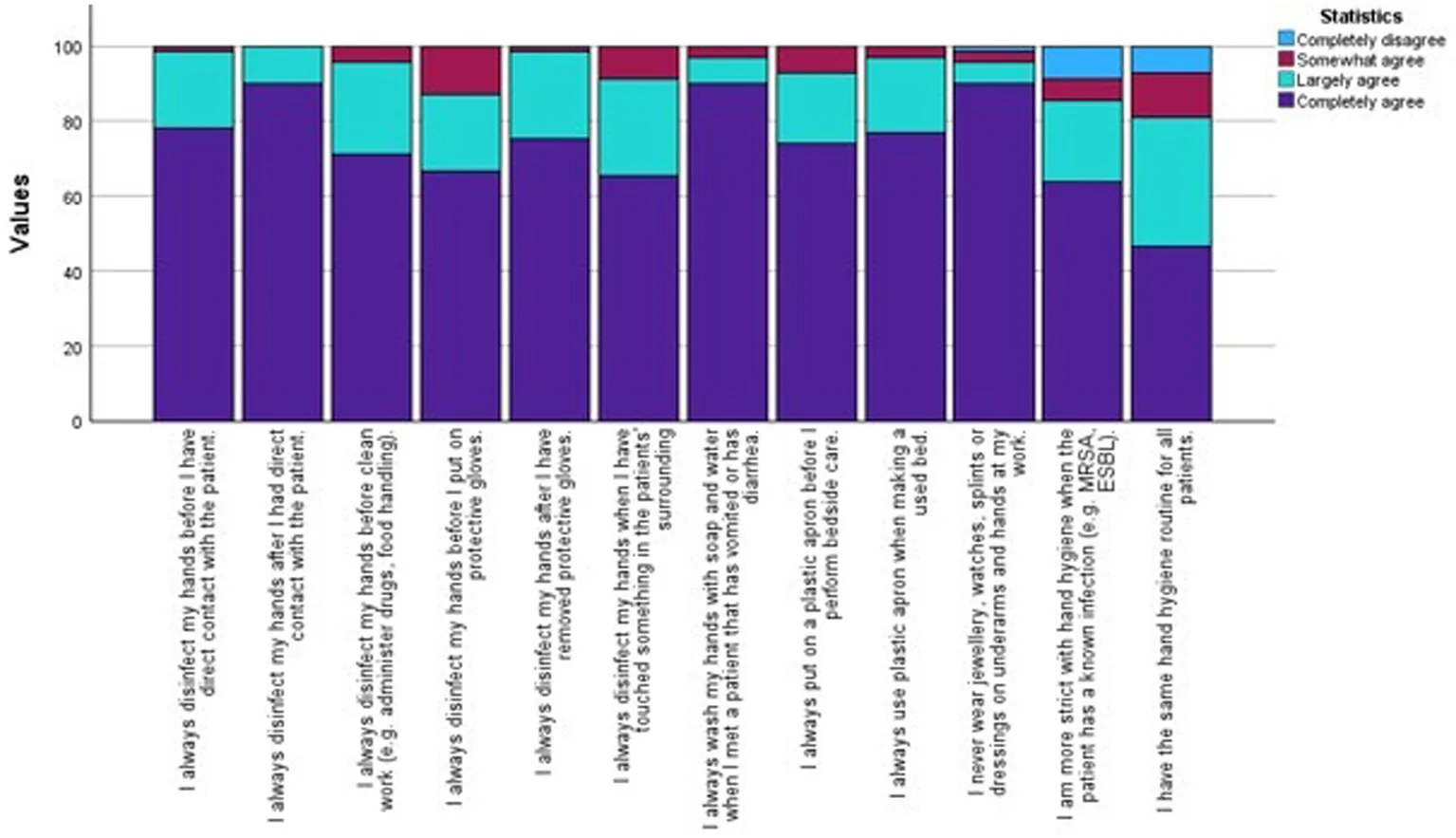
Self-assessment of adherence (*n* = 69).

On the self-assessing scale of adherence to basic hygiene routines, the healthcare personnel assessed themselves to be adherent to 92.1% (SD = 3.3%) of the maximum score. Figure 1 displays the participants’ self-assessment of their adherence to basic hygiene routines. A one-way ANOVA showed no statistically significant differences between the professions regarding the self-assessed adherence score (assistant nurses: *M* = 91.7%, SD = 7.3%; registered nurses: *M* = 93.1%, SD = 8.1%; physicians: *M* = 90.6%, SD = 6.9%, *F*(2, 66) = 2.00, *p* = 0.143, *η*^2^ = 0.057). Furthermore, on the single-item question, 37.7% assessed themselves to always adhere to and 62.3% to often adhere to basic hygiene routines. A separate one-way ANOVA revealed no significant differences among professions regarding this single-item question (assistant nurse: *M* = 3.53, SD = 0.50; registered nurses: *M* = 3.35, SD = 0.48; physicians: *M* = 3.15, SD = 0.38, *F*(2, 66) = 2.92, *p* = 0.061, *η*^2^ = 0.081).

The median knowledge test score was 90.9% of the maximum possible score (IQR = 81.8–97.7) for all included healthcare personnel. A Kruskal–Wallis test revealed a significant difference in knowledge scores across professions, *H* (2) = 14.94, *p* < 0.001. Post-hoc pairwise comparisons using the Bonferroni correction showed that registered nurses (Mdn = 97.7%, IQR = 94.3–100%) had significantly higher knowledge scores than assistant nurses (Mdn = 90.9%, IQR = 81.8–97.7%, adjusted *p* = 0.003) and physicians (Mdn = 88.6%, IQR = 77.3–95.5, adjusted *p* = 0.005).

In the self-assessment of their own knowledge, a quarter (24.6%) of the total sample viewed their own knowledge as very good, 63.8% as good, and 11.6% as fairly good. There were no statistically significant differences between the professions. A one-way ANOVA revealed no statistically significant differences between the professions regarding this self-assessed knowledge score (assistant nurses: *M* = 3.15, SD = 0.53; registered nurses: *M* = 3.24, SD = 0.58; physicians: *M* = 2.85, SD = 0.69, *F*(2, 66) = 2.09, *p* = 0.132, *η*^2^ = 0.059).

The healthcare personnel thought it was very important (88.4%) or important (11.6%) to have knowledge of basic hygiene routines in healthcare. A Kruskal–Wallis test revealed a significant difference in the perceived importance of knowledge across professions, (*H* (2) = 19.13, *p* < 0.001). Post-hoc pairwise comparisons using the Bonferroni correction showed that the importance of knowledge was viewed as significantly higher by registered nurses (100% ‘Very important’) and assistant nurses (92.6% ‘Very important’, 7.4% ‘Important’) compared to physicians (53.8% ‘Very important’, 46.2% ‘Important’; adjusted *p* < 0.001 vs. registered nurses, adjusted *p* = 0.001 vs. assistant nurses.)

### 3.3 Correlations between knowledge on and adherence to basic hygiene routine

For the total sample, there were no significant correlations between the knowledge test scores, the observed adherence, or the self-assessed adherence. However, for the individual professions, a significant negative correlation was found between the knowledge test scores and the single-item self-assessment of adherence among registered nurses (*r_s_* = −0.38, *p* = 0.04). See Table 2.

**Table 2:** Spearman’s rank correlation coefficient between knowledge test score and observed, respectively, self-assessed adherence to basic hygiene routines and self-assessed knowledge.

Variable	Knowledge test score			
	Total	Assistant nurse	Registered nurse	Physician
Observation of adherence	0.18	0.12	−0.10	0.15
Self-assessed score of adherence	0.09	−0.15	0.19	−0.05
Single-item self-assessment of adherence	−0.10	0.12	−0.38*	0.00
Self-assessed knowledge	0.13	0.52**	−0.28	−0.07

*p = 0.04, **p = 0.006.

The self-assessed knowledge showed a significant positive correlation with the single-item self-assessed adherence (*r* = 0.44, *p* < 0.001) in the whole sample, but not with observed adherence or the self-assessed adherence score. Assistant nurses and registered nurses displayed the same pattern, showing a significant positive correlation between self-assessed knowledge and the single-item self-assessed adherence (assistant nurses *r* = 0.41, *p* = 0.032; registered nurses *r* = 0.46, *p* = 0.012), but not with observed adherence or the self-assessed adherence score. For physicians, no significant correlations were found between self-assessed knowledge and either observed or self-assessed adherence. See Table 3.

**Table 3:** Pearson correlation coefficient between self-assessed knowledge and observed, respectively, self-assessed adherence to basic hygiene routines.

Variable	Self-assessed knowledge			
	Total	Assistant nurse	Registered nurse	Physician
Observation of adherence	0.13	0.01	0.04	0.14
Self-assessed score of adherence	0.00	−0.05	−0.09	0.15
Single-item self-assessment of adherence	0.44*	0.41**	0.46***	0.42

*p < 0.001, **p = 0.032, ***p = 0.012.

The correlation between the knowledge test scores and the self-assessed knowledge was non-significant for the whole sample, as well as for registered nurses and physicians. Conversely, assistant nurses exhibited a significant positive correlation (*r_s_* = 0.52, *p* = 0.006). See Table 2. Furthermore, there was a significant positive correlation between the observed adherence and both the self-assessed adherence score (*r* = 0.26, *p* = 0.03) and the single-item self-assessed adherence (*r* = 0.25, *p* = 0.04) for the total sample. For the individual professions, physicians demonstrated a significant positive correlation between observed adherence and the self-assessed adherence score (*r* = 0.61, *p* = 0.027). In contrast, no significant correlations between observed and self-assessed adherence were found for assistant nurses and registered nurses. See Table 4.

**Table 4:** Pearson correlation coefficient between observed adherence and self-assessed adherence.

	Total			Assistant nurse			Registered nurse			Physician		
1 = Observation of adherence; 2 = Self-assessed adherence score; 3 = Single-item self-assessed adherence												
Variable	1	2	3	1	2	3	1	2	3	1	2	3
1. Observation of adherence	–	0.26*	0.25**	–	0.36	0.01	–	−0.06	0.32	–	0.61***	0.51
2. Self-assessed score of adherence		–	−0.06		–	0.21		–	−0.33		–	−0.03
3. Single-item self-assessment of adherence			–			–			–			–

*p = 0.03, **p = 0.04, ***p = 0.027.

### 3.4 Work environment and adherence to hand hygiene routines

**Figure 2:**
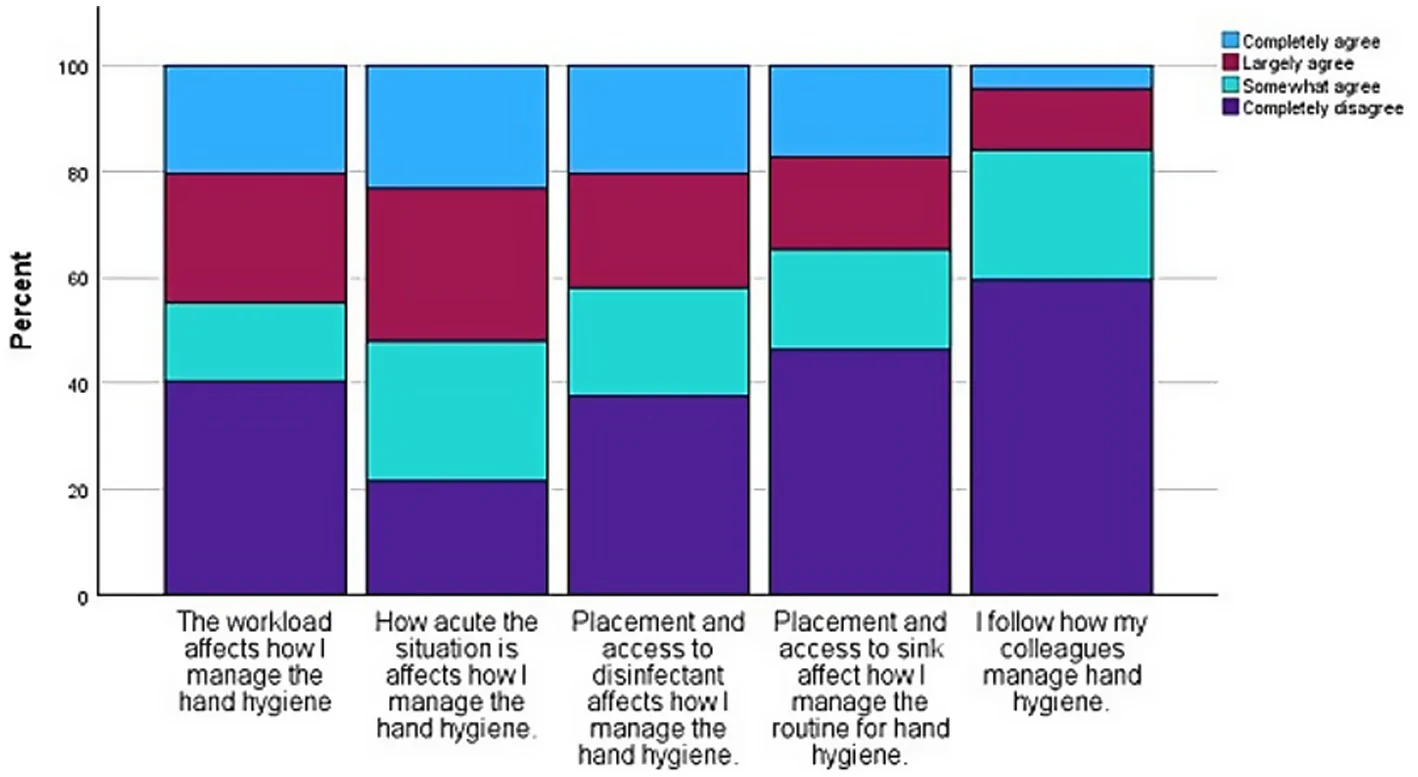
Self-assessment of how work environment affects management of basic hand hygiene (*n* = 69).

Perceptions varied on how workload affected the management of hand hygiene, where 44.9% completely or largely agreed, whereas 40.6% completely disagreed. The majority (52.1%) agreed largely, or completely that acute situations affected their adherence, while a fifth (21.7%) completely disagreed. Concerning the placement of and access to hand disinfectant, 42.0% agreed to a large or full extent that it affected their hand hygiene practices whereas 37.7% completely disagreed. Almost half (46.4%) completely disagreed that the placement of and access to a sink affected their adherence, while 34.8% agreed largely or completely. Furthermore, the majority (59.4%) completely disagreed that they aligned their own hand hygiene behavior with that of their colleagues. See Figure 2.

A one-way ANOVA revealed no significant differences between the professions regarding the total score for the self-assessed impact of the work environment on adherence to hand hygiene routines (assistant nurses: *M* = 14.11, SD = 3.90; registered nurses: *M* = 14.69, SD = 4.18; physicians: *M* = 13.85, SD = 4.47, *F*(2, 66) = 0.24, *p* = 0.790, *η*^2^ = 0.007).

## 4 Discussion

This study examined adherence to basic hygiene routines and knowledge in an infectious disease clinic at a university hospital in Sweden. The present study demonstrates deficiencies in adherence to basic hygiene routines, which is consistent with previous studies indicating that hand hygiene or basic hygiene routines are followed to varying degrees. Values as low as 37% adherence have been documented in earlier research (13). In another study, adherence rates at baseline were similar to those observed here, but after 6 years of regular observations and feedback, adherence increased to 88.5% (22). Yet another study reported 65% adherence at baseline; after an educational intervention, adherence rose to 88%, showing that further education can lead to improved adherence (17). Since the present study was conducted in an infectious disease clinic, there was an assumption that the participants would exhibit higher knowledge and adherence to basic hygiene routines than the levels reported in previous research conducted in other clinics. While adherence in the present study exceeded the baseline adherence reported in previous studies, post-intervention adherence rates in those studies increased to levels higher than observed in the present study (17, 22). Furthermore, a study found that 7 out of 9 healthcare workers in an infectious disease clinic had contact with a colleague after patient contact without disinfecting their hands in between (23). Consequently, these findings demonstrate that working in an infectious disease clinic does not necessarily translate to high adherence to hygiene routines.

In the present study, registered nurses had significantly higher adherence to basic hygiene routines compared with physicians, and significantly higher knowledge compared with both physicians and assistant nurses. Despite this, a significant negative correlation between knowledge level and adherence was found among registered nurses, and no correlation was observed for the other groups. Thus, the findings do not indicate that higher knowledge leads to higher adherence. Several studies contradict these results, reporting that knowledge levels positively influence adherence (15, 22, 24). Conversely, Dowding et al. (25) support the results of the present study, stating that adherence to hygiene routines may remain insufficient despite high levels of knowledge, although a lack of knowledge remains an important factor in non-adherence (7). While knowledge is a fundamental component of clinical practice, research suggests that it does not necessarily translate into behavior. For instance, studies by Silva et al. (26) and Sagar et al. (27) demonstrate no statistically significant correlation between healthcare workers’ knowledge levels and their adherence to hand hygiene routines. This ‘knowledge-practice gap’ indicates that providing education alone is not sufficient to ensure compliance. Instead, adherence appears to be more closely linked to situational and environmental factors.

The present study showed that the majority reported improving their hand hygiene when the patient was a known carrier of antibiotic-resistant bacteria. It is known that antibiotic-resistant bacteria are a growing concern; for instance, an outbreak of MRSA previously occurred in a Swedish hospital maternity ward (28). This outbreak was successfully contained due to improved cleaning and stricter adherence to basic hygiene routines. In line with this, education about hygiene routines, their importance, and the consequences of non-adherence has been shown to increase both the use of hand disinfectant and adherence to hygiene routines (14, 17, 18).

The observational results revealed differences in adherence between the professions. This aligns with previous research by Karaaslan et al. (13), Bredin et al. (29) and Wetzker et al. (30), all of which report that registered nurses demonstrate better hand hygiene adherence compared with physicians. Physicians who receive encouragement and feedback from registered nurses tend to follow hand hygiene practices to a higher degree than when such daily reminders and support are absent (31). Since adherence to basic hygiene routines tends to increase when feedback and reminders are provided, adherence could be enhanced by encouraging healthcare workers to actively observe and provide peer feedback regarding adherence to basic hygiene routines. The findings of Ojanperä et al. and Rezk et al. (22, 32) further reinforce the role of feedback in improving adherence. However, McCauley et al. (7) address a potential barrier, noting that medical staff might not always be receptive to feedback from nurses due to institutional hierarchies. A solution to this may lie in the study by Linam et al. (33), where the ‘Speaking Up for Safety Program’ was shown to improve hand hygiene adherence. Thus, through leadership education, knowledge about hand hygiene, cultural change, and addressing known barriers to speaking up, hand hygiene adherence improved among both nurses and physicians. Hagel et al. (34) also report that healthcare workers possess good knowledge of hand hygiene but lack motivation to adhere when supervision is absent.

The knowledge test revealed significant differences between assistant nurses, registered nurses, and physicians. Registered nurses had the highest level of knowledge about basic hygiene routines, and physicians the lowest. These results partly reflect the extent to which staff at the infectious disease clinic have had access to internal training on basic hygiene routines. Consequently, these figures underscore the need to ensure that all professions, including physicians, are provided equal opportunities to participate in and complete training on basic hygiene routines. Consistent with the findings of the present study, physicians performed worse than nurses on the knowledge test in a study by Yousif et al. (35). This may partially explain why physicians exhibit lower adherence to basic hygiene routines compared with registered nurses. However, the absence of a significant correlation between knowledge and adherence contradicts this explanation. Furthermore, the results of the present study and the study by Yousif et al. (35) differ from those of Despo et al. (36), who found that physicians generally possessed the highest levels of hand hygiene knowledge and assistant nurses the lowest.

Among registered nurses, there was a negative correlation between knowledge test scores and the single-item self-assessed adherence. This might suggest that registered nurses with higher levels of knowledge become more self-critical and therefore assess their adherence as lower than those with lower knowledge levels, which may lead to an overestimation of the adherence in the latter group. However, there was no significant correlation between knowledge and the multi-item self-assessed adherence questionnaire, suggesting that this interpretation should be made with caution. A significant association was observed between self-assessed knowledge of basic hygiene routines and performance on the knowledge test among assistant nurses. This indicates that assistant nurses who reported having good knowledge also performed well on the test, demonstrating accurate self-awareness. For registered nurses and physicians, however, negative correlations were found, although neither was significant. This suggests a possible tendency toward overestimation of knowledge among these professions, which has also been described by Piras et al. (37) and Żółtowska et al. (38).

The present study showed variation in how healthcare personnel perceived the impact of workplace factors on adherence to hygiene routines, with some participants reporting that these factors affected their adherence and others reporting little or no influence. Previous studies indicate that stress, high workload, time constraints, lack of motivation, and negative attitudes adversely affect adherence to hygiene routines (14–16). On the other hand, all registered nurses in the study indicated that they considered adherence to basic hygiene routines to be very important, which may contribute to higher motivation. This attitude is reflected in their adherence levels, as registered nurses performed better than both assistant nurses and physicians. Similarly, Karaaslan et al. (13), Ahmadipour et al. (14), and Al-Qahtani (15) report how attitudes and motivation influence hand hygiene compliance. This should be taken into account when designing educational interventions on basic hygiene routines. Additionally, Smiddy et al. (39) discuss how workplace culture affects motivation to follow guidelines.

### 4.1 Strengths and limitations

Since this was a cross-sectional study, no conclusions can be drawn about cause and effect from the results. The study was conducted at a single infectious disease clinic and included a relatively small sample, particularly among physicians, which limits the ability to detect small differences and associations. Consequently, non-significant findings should be interpreted with caution. Based on the sensitivity analysis, the available sample size was sufficient primarily to detect moderate-to-large correlations, as well as effects between the professional groups. However, the sample included all three professional groups in proportions broadly corresponding to their representation within the participating wards. Consistency with previous internal measurements conducted continuously across all wards strengthens the credibility of the observations. The Hawthorne effect may have influenced the study results, as participants were informed that observations would take place during their shift. Therefore, the observed adherence may overestimate adherence under routine conditions. Nevertheless, non-adherence to basic hygiene routines was still observed despite participants’ awareness of being observed. Although the observers used a standardized protocol and reached consensus on uncertain observations during data collection, no formal assessment of inter-rater reliability was performed. This should be considered when interpreting the observational findings. Furthermore, a previously tested knowledge test and self-assessment questionnaire were used, which may have strengthened the reliability and validity of the survey. Moreover, internal reliability tests yielded acceptable to good internal consistency. Initially, internal reliability testing indicated lower than desirable internal consistency (40) for the self-assessment of adherence scale (*α* = 0.652). Psychometric evaluation identified one item concerning stricter hand hygiene when caring for patients with known infections, such as MRSA or ESBL, as problematic. The item showed poor item-total correlation, and its removal improved Cronbach’s alpha. Conceptually, agreement or disagreement with this statement could not be interpreted unequivocally as reflecting higher or lower adherence to standard precautions, suggesting that the item may capture a construct different from general adherence. Consequently, the item was excluded from the total adherence score. This should be considered when interpreting the findings, and the item may warrant revision in future versions of the questionnaire. Another limitation is that the term “hand hygiene” was used instead of “basic hygiene routines” in the work-environment scale, which may have affected the responses to these questions.

### 4.2 Conclusion

Although healthcare professionals demonstrated good knowledge and generally positive self-assessed adherence, observed adherence to hygiene routines was suboptimal and varied between professions. Registered nurses achieved higher scores on the knowledge test and demonstrated greater adherence than assistant nurses and physicians. The lack of correlation between knowledge and adherence suggests that factors beyond knowledge, such as organizational culture and behavioral aspects, are important. Targeted interventions focusing on behavioral change, feedback, and interprofessional collaboration are needed to improve adherence and reduce HAIs. Further studies mapping the factors that increase adherence to basic hygiene routines would be valuable; preferably, through a large-scale study involving more departments. An intervention study evaluating, for example, education or motivational measures in different subgroups would also be informative. Such studies could provide greater insight into how adherence can best be enhanced.

## Data Availability

The datasets generated and analyzed during the current study are not publicly available due to concerns regarding participant anonymity but are available from the corresponding author upon reasonable request.
